# Experimental and Numerical Investigations on the Flow Characteristics within Hydrodynamic Entrance Regions in Microchannels

**DOI:** 10.3390/mi10050317

**Published:** 2019-05-11

**Authors:** Haiwang Li, Binghuan Huang, Min Wu

**Affiliations:** National Key Laboratory of Science and Technology on Aero Engines Aero-thermodynamics, The Collaborative Innovation Middle for Advanced Aero-Engine of China, School of Energy and Power Engineering, Beihang University, Beijing 100191, China; 09620@buaa.edu.cn (H.L.); Huangbh@buaa.edu.cn (B.H.)

**Keywords:** microchannel, velocity profile, microscopic particle image velocimetry (micro-PIV), entrance length

## Abstract

Flow characteristics within entrance regions in microchannels are important due to their effect on heat and mass transfer. However, relevant research is limited and some conclusions are controversial. In order to reveal flow characteristics within entrance regions and to provide empiric correlation estimating hydrodynamic entrance length, experimental and numerical investigations were conducted in microchannels with square cross-sections. The inlet configuration was elaborately designed in a more common pattern for microdevices to diminish errors caused by separation flow near the inlet and fabrication faults so that conclusions which were more applicable to microchannels could be drawn. Three different microchannels with hydraulic diameters of 100 μm, 150 μm, and 200 μm were investigated with Reynolds (Re) number ranging from 0.5 to 50. For the experiment, deionized water was chosen as the working fluid and microscopic particle image velocimetry (micro-PIV) was adopted to record and analyze velocity profiles. For numerical simulation, the test-sections were modeled and incompressible laminar Navier–Stokes equations were solved with commercial software. Strong agreement was achieved between the experimental data and the simulated data. According to the results of both the experiments and the simulations, new correlations were proposed to estimate entrance length. Re numbers ranging from 12.5 to 15 was considered as the transition region where the relationship between entrance length and Re number converted. For the microchannels and the Reynolds number range investigated compared with correlations for conventional channels, noticeable deviation was observed for lower Re numbers (*Re* < 12.5) and strong agreement was found for higher Re numbers (*Re* > 15).

## 1. Introduction

Microchannels draw attention of researchers for their widespread application in microdevices, such as microheat-exchangers [1,2,3,4,5,6] and micromixers [7,8,9,10,11,12]. Different from channels in conventional size, microchannels have larger specific surface areas and higher heat and mass transfer rates, hence better performance. For further development of these microdevices, it is necessary to understand the flow characteristics in microchannels.

Generally, microchannels applied in microdevices are extremely short, meaning that hydrodynamic developing flow may dominate a significant proportion of the whole flow region. In the developing flow region, which is termed as the entrance region, the velocity profile of the flow continuously alters and eventually changes into a constant pattern. The length of this region is addressed as the hydrodynamic entrance length. Generally, the hydrodynamic entrance length can be defined as the length from the inlet of a channel to a certain location in the channel where the maximum local velocity has attained 99% of its fully developed value [13]. This is because after attaining this value, the fluid requires an extra considerably long distance to be shaped into a fully developed flow pattern. Therefore, for engineering applications, this 99% criterion is more applicable to defining the entrance length and the error is acceptable. It is considered that there is distinction between transport properties for the entrance region and those for the fully developed region. Attention should be paid to the summary of conventional correlations for friction factor, heat transfer coefficient, and laminar to turbulent transition from fully developed flow, so that the measurement points are sufficiently far away from the inlet (Yun et al. [14] indicated that ratios of channel length to hydraulic diameter ($L/D_{h}$) should greater than 70. Rohsenow and Hartnett [15] stated that the length for laminar flow to achieve full development in a smooth duct can be defined as $\frac{L/D_{h}}{Re\cdot Pr}\geq 0.05$ and the length is much shorter for turbulent flow), so that the effects of the entrance region can be neglected. However, for flow in microchannels which are insufficient in length, error may be caused when correlations for fully developed flow are applied. Therefore, it is of great importance to research the entrance length in microchannels. 

Hydrodynamic entrance length for conventional scale channels has been explored by numerous scholars, both experimentally and numerically. Experimental studies for conventional scale ducts have been conducted by groups such as Beavers et al. [16], Sparrow et al. [17], and Goldstein and Kreid [18]. Atkinson et al. [19] and Chen [20] investigated the entrance length for conventional scale flows in circular pipes and between parallel plates at low Reynolds numbers numerically and drew correlations which correspond well with experimental data. Two forms of correlation were proposed in their works, shown as Equation (1) and Equation (2), respectively, where $L_{e}$ is hydrodynamic entrance length, $D_{h}$ is hydraulic diameter, and *Re* is Reynolds number. Coefficients *A*, *B*, and *C* are listed in Table 1.

(1) $$\frac{L_{e}}{D_{h}}=A+B\cdot Re$$

(2) $$\frac{L_{e}}{D_{h}}=\frac{A}{B\cdot Re+1}+C\cdot Re$$

However, compared with channels in conventional scale, due to the reduction in the characteristic scale, the impact of viscous force, surface tension, and electricity force is more important on flow in microchannels [21,22,23]. Surface roughness, wettability, surface charge, and other factors also affect the flow pattern in the meantime [24,25,26]. Moreover, the measurement uncertainty of the results obtained in microchannels experiments is much larger than that in conventional channels, which results in inconsistency among the conclusions of flow characteristics in microchannels drawn by different researchers. Therefore, it is still controversial whether the classical fluid theory is applicable to microfluidic systems and adopting new technical approaches is important.

Microscopic particle image velocimetry (micro-PIV) is a new and effective experimental technique which could be used to analyze flow pattern and hydrodynamic entrance length in microchannels more directly and clearly. Comprehensive flow data, including local velocity and velocity profiles of cross-sections, can be obtained through micro-PIV with less disturbance on the flow field and entrance length could be confirmed based on these data. Many achievements have been obtained regarding investigating flow field and the transition from laminar to turbulent flow in microchannels with micro-PIV. For example, Zhang et al. [27] conducted experiments to research flow characteristics of deionized water and kerosene in microchannels with diameters ranging from 50 to 254 μm under very low Reynolds number conditions ($10^{-5}<Re<10^{-2}$). Micro-PIV was applied to measure the velocity distribution inside the microchannels and the result was in strong agreement with the value calculated by the Navier–Stokes equation.

Lee et al. [28] carried out a series of micro-PIV experiments using deionized water as working fluid. Deionized water flowed through a rectangular cross-section acrylic microchannel which was 120 mm in length, 690 μm in height, and 260 μm in width. The Reynolds numbers explored in the experiments varied from 250 to 2100. Images were taken at different locations along the length of the microchannel to obtain averaged correlation velocity profiles. The results of the analysis showed that the entrance lengths were shorter than predicted, according to correlations proposed by Shah and London [19]. The authors explained that the fluid had been pre-developed in the pipe before entering the microchannel, which reduced the length of entrance by 45%. 

Lee and Kim [29] investigated the influence of different inlet configurations on the length of the entrance region in microchannels. Channels were etched on silicon wafer, which were 30 mm in length, 58 μm in depth, and 100 μm in width. Six different inlet configurations with curvature radii ranging from *r* = 20 μm to 150 μm were fabricated and their influence on downstream velocity was investigated in their study with micro-PIV. Deionized water was injected into channels at about *Re* = 1. Conclusions were drawn that rounding the inlet corner would smooth incoming flow and reduce flow resistance and the entrance length for microchannels was much smaller compared with conventional channels.

Moreover, some scholars have drawn empirical correlations for entrance length in microchannels. Hao et al. [30] observed velocity profiles at various positions along the length of the channel to analyze the developing process of water flow in trapezoidal silicon microchannels with a hydraulic diameter of 237 μm; the Reynolds numbers varied from 50 to 1200. Micro-PIV was adopted to obtain stream-wise centerline velocity variance in microchannels, which illuminated that the hydraulic entrance length ($L_{e}$) was about $\frac{L_{e}}{D_{h}}=0.08–0.09Re$.

Ahmad and Hassan [31] investigated the entrance length for deionized water flow in microchannels experimentally with micro-PIV. The cross-sections of the microchannels were square with the hydraulic diameters of 100, 200, and 500 μm. Four different Reynolds numbers (*Re* = 0.5, 5, 50, and 200) were tested. A comparison was made between their results and conventional correlations of entrance length for macro-scale channels and inconsistency had been observed for some Reynolds numbers investigated by them. They modified the coefficients and proposed new empirical correlations to predict the entrance length for square cross-section microchannels.

Although a great deal of research has been done (Table 2), investigations on hydrodynamic entrance length for laminar in microchannels are still limited and there is controversy among different groups’ conclusions. Lee and Kim [29] indicated that the entrance length for microchannels was much shorter compared with conventional channels, while the results of Hao et al. [30] and Ahmad et al. [31] showed that using conventional correlations for macro-scale channels would underestimate the entrance length of microchannels. Further research is necessary to illuminate the influence factors on hydrodynamic entrance length in microchannels.

In this work, hydrodynamic entrance regions in microchannels were investigated both experimentally and numerically. Compared with previous research, a new inlet configuration was elaborately designed in a more common pattern for microdevices to diminish errors caused by separation flow near the inlet and fabrication faults, allowing conclusions which were more applicable to microchannels to be drawn. In the experiment, micro-PIV was used to obtain local velocity and flow profiles in the mid-depth plane of laminar deionized water flow in microchannels. The microchannels were of square cross-section, with hydraulic diameters of 100 μm, 150 μm, and 200 μm. Reynolds numbers tested in experiments varied from 0.5 to 50. In numerical simulation, microchannels investigated in experiment were modeled and incompressible laminar Navier–Stokes equations were solved with ANSYS CFX 15.0 (ANSYS Inc, Pittsburgh, PA, USA). The parameters investigated in the simulation were the same as those in the experiment.

## 2. Experimental Description

### 2.1. Microchannel Fabrication

**Figure 1:**
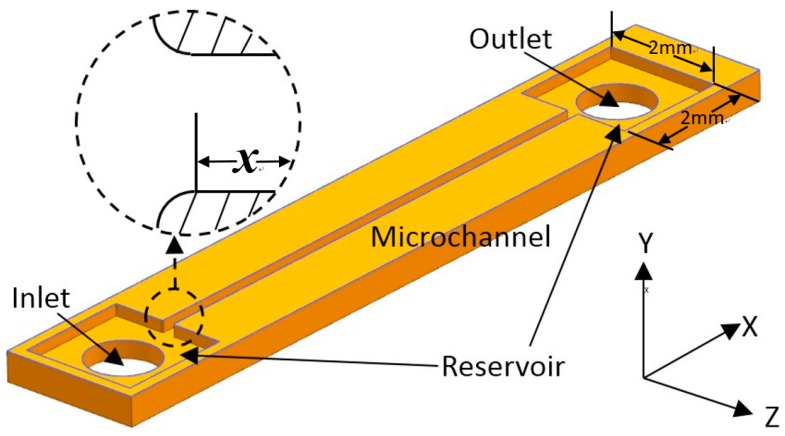
Design of the square cross-section microchannels.

**Figure 2:**
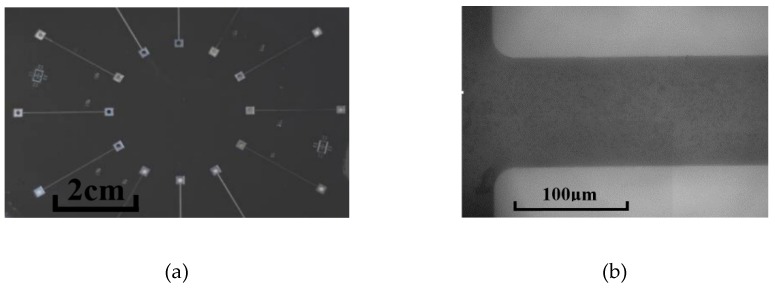
Images of actual microchannels. (**a**) Microchannels etched on silicon wafer. (**b**) Microscope view of one of the microchannels (*D_h_* = 100 μm).

The design of the microchannels is illustrated in Figure 1. Instead of connecting the inlet and outlet of a channel to tubes directly, both ends of the channel were connected to expanding segments with a width of 2 mm, performing as reservoirs. The reservoirs could diminish flow fluctuation and avoid the effect of pre-developing when the inlet of the channel was connected to the tube directly. The microchannels were 100 μm, 150 μm, and 200 μm in hydraulic diameters ($D_{h}$) with a square cross-section (height/width = 1) and all the microchannels were 20 mm in length. Considering that it was difficult to fabricate an exactly right-angled inlet for microchannels because of unavoidable wearing away during the etching process, quadrants with radii of 20 μm were designed to supplant the right-angle corner of the inlets of all the microchannels and the inlet of the channel was defined as *x* = 0. The patterns of the microchannels were etched on one side of a double-sided polished silicon wafer and the bottom surfaces of the channels and reservoirs were in the same plane, which is more common in microdevices. With this configuration, the inlet velocity profile was considered to be symmetrical and separation flow near the inlet would be diminished. Then, two holes for the inlet/outlet tubes were etched in the middle of the two reservoirs. The side with the channels was bonded with a glass plate, while inlet and outlet tubes were glued on the other side. The images of the actual microchannels are shown in Figure 2.

### 2.2. Experimental Setup

**Figure 3:**
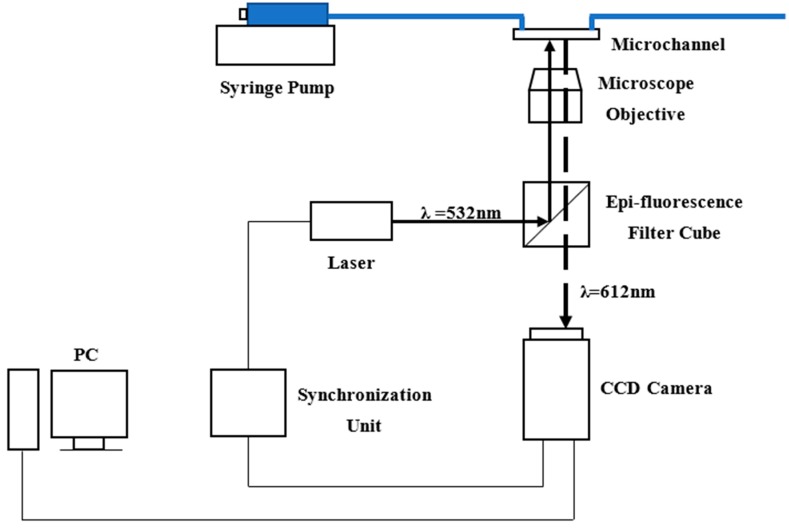
Schematic diagram of experimental setup.

The experimental system consisted of syringe pump and a micro-PIV system and is illustrated in Figure 3. Particles of 1 μm in diameter with a specific gravity of 1.05 were used in experiments. The particle concentration in the deionized water was about 0.02% by volume. The Reynolds numbers were controlled by a syringe pump, which was programmed into a desired flowrate and operated to inject deionized water into microchannels. Reynolds numbers were calculated by Equation (3)

(3) $$Re=\frac{QD_{h}}{\nu A},$$

where *Q* is flowrate, $D_{h}$ is the hydraulic diameter, ν is the kinematic viscosity which depends on the room temperature (25 °C for all the experiments in this work) and assumed to be constant, and *A* is the cross-section area of microchannel. 

**Figure 4:**
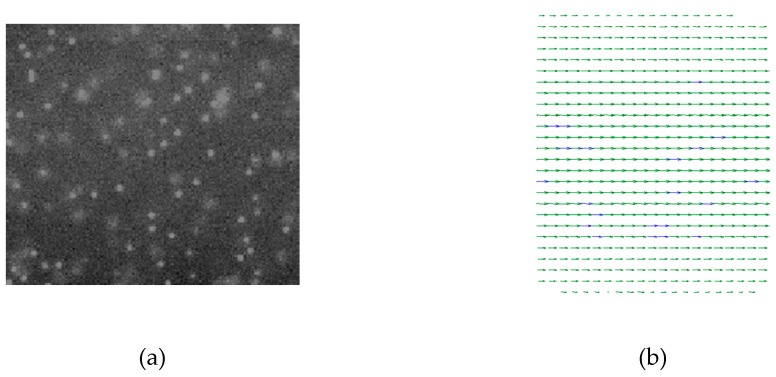
Image of particles and velocity vectors. (**a**) Particles illuminated by laser. (**b**) Velocity vectors in microchannel.

Velocity profiles in microchannels were measured by the micro-PIV system. A dual-pulsed Nd: YAG laser light (wave length is 532 nm), controlled by computer software, was passed through a beam expander assembly and directed into the objective lens of the inverted microscope. Particles mixed in deionized water were illuminated by laser, then fluorescence was excited and the light emitted by the particles passed through the objective lens and the dichroic mirror. The images were recorded with an interline transfer CCD camera with a 2072×2072 pixel array and 12-bit read-out resolution. The instantaneous velocity vectors in the microchannels were derived from a cross-correlation algorithm and an adaptive-PIV algorithm and 100 pairs of frames were averaged to obtain the mean velocity profiles. The images of the particles and the velocity vectors are shown in Figure 4.

### 2.3. Experimental Uncertainty

The uncertainty in the experiments may derive from errors in flowrate, hydraulic diameters of microchannels, and the micro-PIV system. The syringe pump employed a stepper motor and worm gear to control the flowrate. Because of the operation of the stepper motor and the friction of the plunger, the highest uncertainty would be ~1–2% when the flowrate was at its lowest. The uncertainty in hydraulic diameters of microchannels was 3% for 100 μm, 1.53% for 150 μm, and 0.75% for 200 μm. The uncertainty in the flowrate and the hydraulic diameters of microchannels caused an uncertainty of 3.6% at most in Re. The micro-PIV data obtained in the fully developed region were compared with results of the Navier–Stokes equation, which showed that the uncertainty was less than 5%. 

There was also random error in the measurement process of micro-PIV, due to Brownian motion as well as interrogation and resolution. Brownian motion refers to apparent random particle movements because of fluid-particle interaction on a molecular level if the particle is sufficiently small. The particle was so small that unbalanced collision between particle and fluid molecules would result in random movement. However, increase in particle size or fluid velocity would decrease the significance of Brownian motion. Error due to Brownian motion can be estimated by the equation provided by Santiago et al. [32]. The largest error due to Brownian motion in these experiments was 0.68%, when *Re* = 0.5 for the 100 μm channel.

The random error on account of interrogation and resolution derives from the uncertainty in particle displacement in the images. According to Santiago et al. [32], the uncertainty in particle displacement could be estimated by assuming that particle displacements measured in experiments are within 1/10^th^ of a seed particle image. The image diameter for a particle in the object plane can be estimated by

(4) $$d_{s}=1.22\left(1+M\right)\lambda NA^{-1},$$

(5) $$d_{e}={\left(M^{2}d_{p}^{2}+d_{s}^{2}\right)}^{1/2},$$

where $d_{s}$ is the characteristic diameter of the point-spread function, *M* is the magnification, *NA* is the numerical aperture of objective lens, λ is the wavelength of the fluorescent light emitted by the particle, $d_{e}$ is the image diameter of particle, and $d_{p}$ is the diameter of particle. The objective lens applied in experiments was of 40× magnification, *NA* = 0.55, and the diameter of particle was 1 μm, making $d_{e}$ 1.71 μm, which projected back into flow coordinates, therefore the particle displacement should be 0.171 μm. The maximum particle displacement was approximately 4 μm between laser pulses, resulting in an experimental uncertainty of 4.3%.

## 3. Experimental Results and Discussion

### 3.1. Developing Velocity Profiles

**Figure 5:**
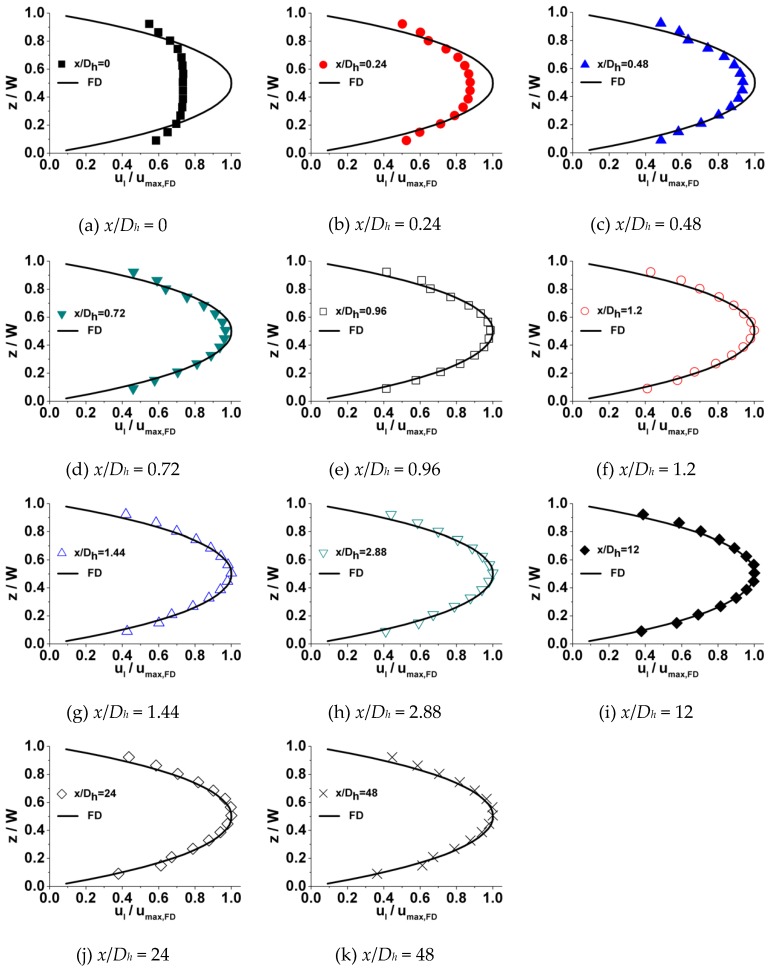
Profiles at different locations along the microchannel length in a 200 μm microchannel (*Re* = 20). (*x*/*D_h_* is the dimensionless distance away from the inlet).

A typical developing velocity profile at a series of incremental axial locations along the microchannel length is shown in Figure 5. In these plots, local velocity ($u_{l}$) is normalized with the theoretical maximum centerline fully developed axial velocity ($u_{max,FD}$) on the x-axis ($u_{l}/u_{\max,\mathrm{FD}}$), while the location in spanwise direction (z) is normalized with the microchannel width (W) on the y-axis (z/W) for all the data. Theoretical fully developed velocity distributions (FD) estimated by the Navier–Stokes equation [33] are also shown in each plot. The physical mechanism of flow development in the microchannel can be observed from these plots. As fluid enters the microchannel from the reservoir, the velocity of the fluid, which is contacted with the inner surface of walls, reduces to zero immediately. On account of viscosity, this effect diffuses from the inner surface of the walls towards the central zone of channel, causing fluid adjacent to the wall to be decelerated. However, in the central zone of the channel, the fluid has not been affected yet. Due to incompressibility and continuity, fluid is displaced from the zone near walls towards the central zone of channel, resulting in the fluid in this zone being accelerated. The cooperation of deceleration adjacent to the inner surface of walls and acceleration near the central zone of channel continually proceeds along the length of the channel, transforming the velocity profile in the channel. Eventually, the velocity profile is altered into the shape of a parabola and no further change is observed at the downstream zone; the flow is considered to be fully developed. 

**Figure 6:**
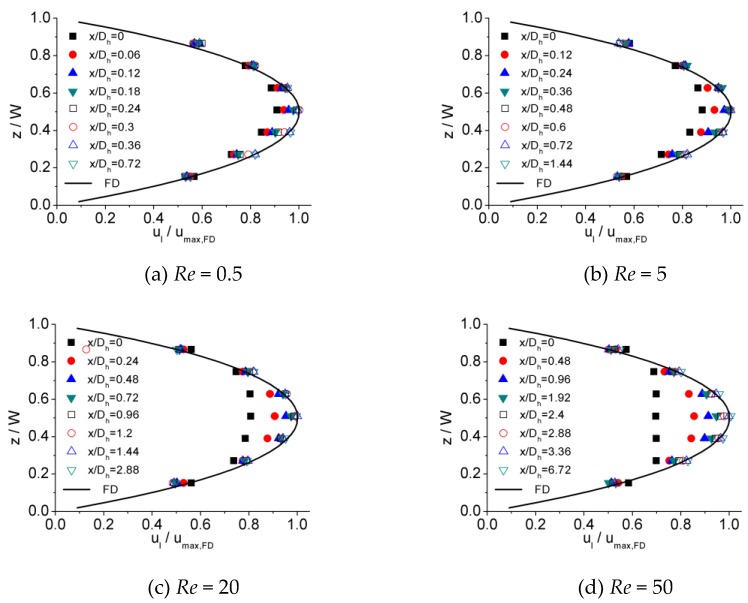
Developing velocity profiles for the 100 μm channel at Re of (**a**) 0.5, (**b**) 5, (**c**) 20, and (**d**) 50.

**Figure 7:**
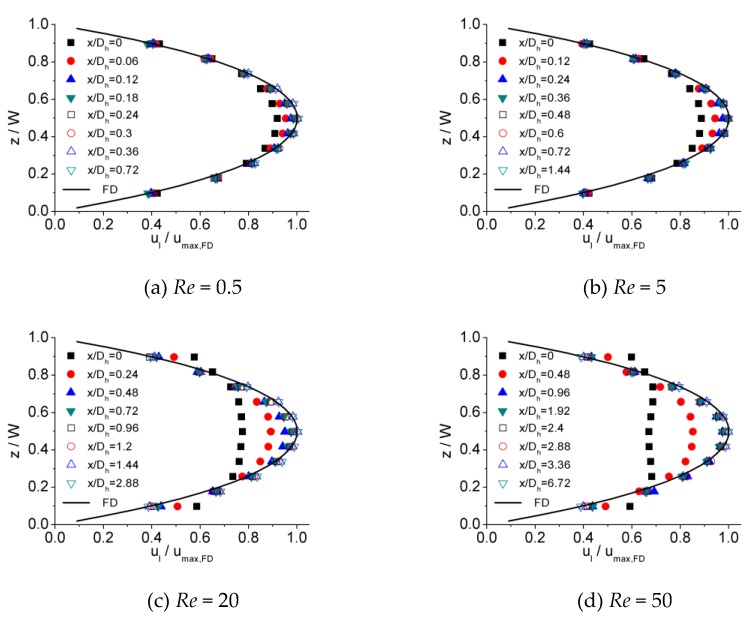
Developing velocity profiles for the 150 μm channel at Re of (**a**) 0.5, (**b**) 5, (**c**) 20, and (**d**) 50.

**Figure 8:**
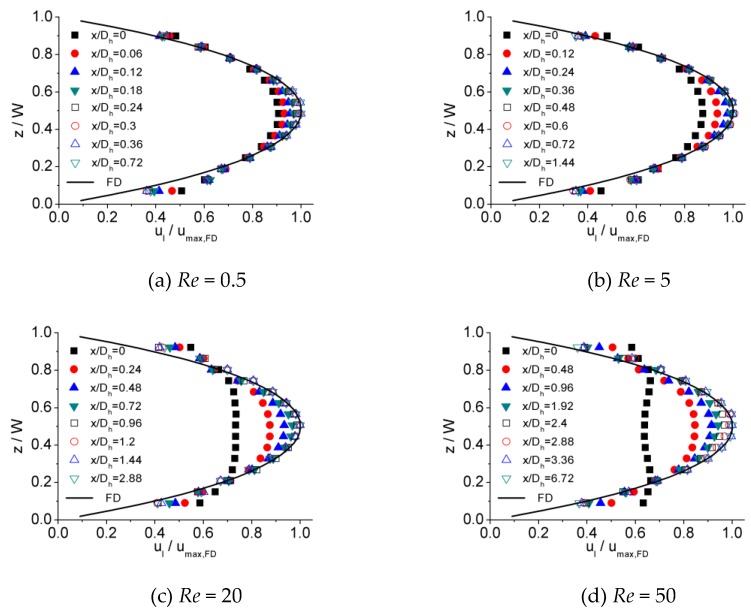
Developing velocity profiles for the 200 μm channel at Re of (**a**) 0.5, (**b**) 5, (**c**) 20, and (**d**) 50.

Figure 6, Figure 7 and Figure 8 illustrates the process of velocity profile alteration at different Re numbers in all the three microchannels. It can be observed from plots that as Re number increases, the values of $u_{cl}/u_{cl,FD}$ at the channel inlet decease for all three microchannels. Moreover, for lower Re numbers, the velocity profiles near the inlet are already in a shape approximated to fully developed and the value of $u_{cl}/u_{cl,FD}$ is relatively high, while for higher Re numbers the velocity profiles near the inlet are flatter and the values of $u_{cl}/u_{cl,FD}$ are smaller, hence a longer distance is requested to alter the velocity profile until it is fully developed. 

### 3.2. Velocity Development Along Centerline

**Figure 9:**
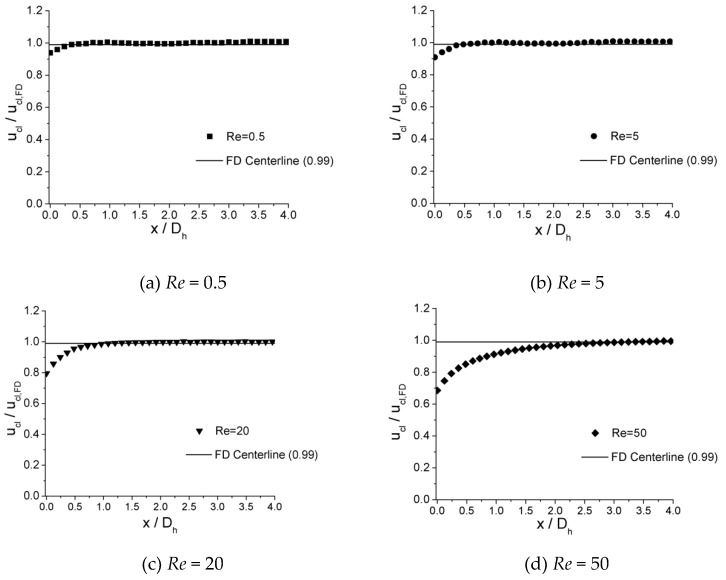
Centerline velocity development for the 100 μm channel at Re of (**a**) 0.5, (**b**) 5, (**c**) 20, and (**d**) 50.

**Figure 10:**
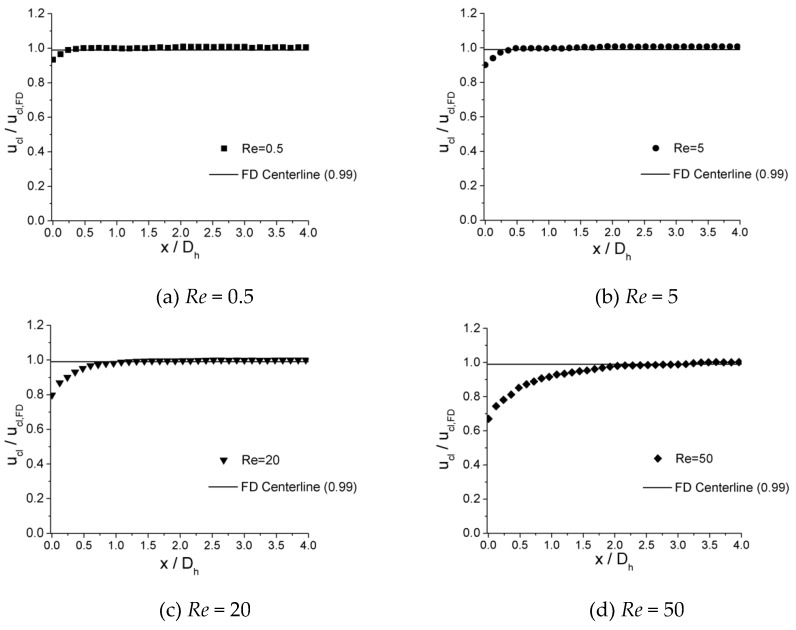
Centerline velocity development for the 150 μm channel at Re of (**a**) 0.5, (**b**) 5, (**c**) 20, and (**d**) 50.

**Figure 11:**
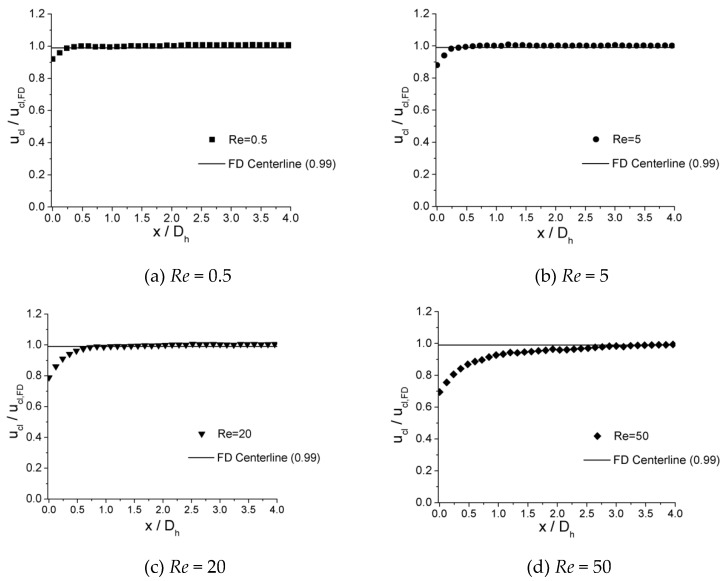
Centerline velocity development for the 200 μm channel at Re of (**a**) 0.5, (**b**) 5, (**c**) 20, and (**d**) 50.

The velocity development at incremental axial location along the centerline of microchannels is shown in Figure 9, Figure 10 and Figure 11 for 100 μm, 150 μm, and 200 μm, respectively. In the plots, local centerline velocities ($u_{cl}$) are normalized with the centerline fully developed axial velocity ($u_{cl,FD}$) on the y-axis ($u_{cl}/u_{cl,FD}$), while the axial distances from inlet of channel (x) are normalized with the hydraulic diameter $D_{h}$ on the x-axis (x/D). 

It is illustrated from the plots that, in all three microchannels, similar traits are shown for the value of $x/D_{h}$ where $u_{cl}/u_{cl,FD}\approx 0.99$ is attained. For lower Re numbers, such as *Re* = 0.5, the values of $x/D_{h}$ are relatively small, which are 0.310 for 100 μm, 0.300 for 150 μm, and 0.325 for 200 μm. As the Re number increases, the values of $x/D_{h}$ increase. When *Re* = 5, the values of $x/D_{h}$ are 0.410 for 100 μm, 0.400 for 150 μm, and 0.420 for 200 μm. As the Re number increases from 0.5 to 5 by 10 times, $x/D_{h}$ increases from approximately 0.32 to 0.42 by only 30%. For lower Re numbers (*Re* < 12.5), the length of entrance region shows less dependence on the Re number. However, for higher Re numbers (*Re* = 15–50), the values of $x/D_{h}$ depend significantly on the Re number. When *Re* = 15, the values of $x/D_{h}$ are 0.850 for 100 μm, 0.833 for 150 μm, and 0.845 for 200 μm. As the Re number increases to 50, the values of $x/D_{h}$ are 3.200 for 100 μm, 3.133 for 150 μm, and 3.150 for 200 μm. For this range of Re number (*Re* = 15–50), the relationship of $x/D_{h}$ versus Re could be approximately considered as a linear relationship.

### 3.3. Correlations of Hydraulic Entrance Length

**Figure 12:**
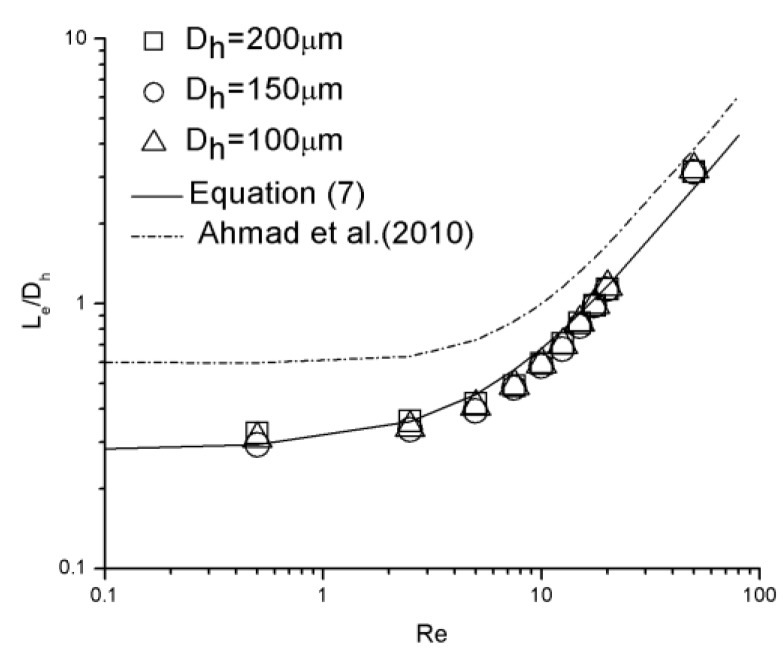
Dimensionless hydraulic entrance length comparison between experimental data and proposed correlations.

Figure 12 illustrates the dimensionless hydraulic entrance length ($L_{e}/D_{h}$) versus the Re number for 100 μm, 150 μm, and 200 μm channels, compared with correlation proposed by Ahmad et al. [31] as

(6) $$\frac{L_{e}}{D_{h}}=\frac{0.6}{0.14Re+1}+0.0752Re.$$

There are 10 data points (*Re* = 0.5, 2.5, 5, 7.5, 10, 12.5, 15, 17.5, 20, and 50) for each channel. Due to poor agreement between the experimental data and this correlation, the form of Equation (2) proposed by Chen [20] was adopted and the new correlation is given as

(7) $$\frac{L_{e}}{D_{h}}=\frac{0.28}{0.1Re+1}+0.0537Re.$$

The correlations fits the data to within 15.2% for Equation (7).

It can be realized that the relationship of dimensionless hydraulic entrance length versus Re number shows different characteristics for lower Re numbers (*Re* < 12.5) and higher Re numbers (*Re* > 15). There is a nonlinear relationship between hydrodynamic entrance length and Reynolds number for *Re* < 12.5, which is converted into a linear relationship as Reynolds number increases (*Re* > 15). To achieve better estimation, the correlation can be separated into two segments. The modified correlation is given as

(8) $$\frac{L_{e}}{D_{h}}=\{\begin{array}{c} \frac{0.3}{0.1Re+1}+0.043Re\;(Re<12.5)\\ \;0.06Re\;(15<Re<50) \end{array}.$$

**Figure 13:**
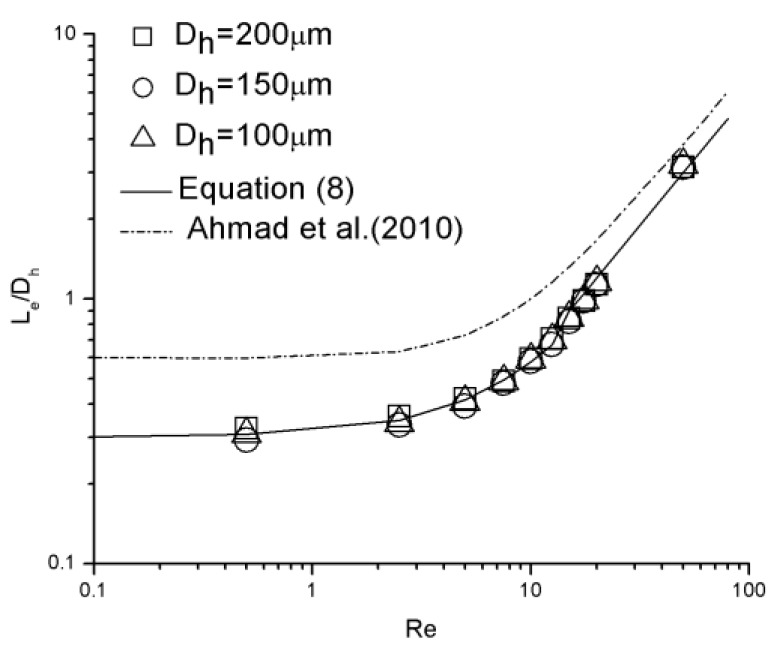
Dimensionless hydraulic entrance length comparison between experimental data and modified correlations.

Because of limited data points, the Re number range from 12.5 to 15 is considered a transition region; both nonlinear and linear correlations could be applied. A comparison between the experimental data and Equation (8) is shown in Figure 13. There is a strong agreement between the data and the correlation with the error smaller than 6.8%. 

It should be emphasized that the coefficients for a correlation in the form of Equation (2) must satisfy the constraint condition A·B<C. Under this condition, the minimum value of the correlation function locates at a point where *Re* < 0, so that the value of the correlation function would monotonously increase as the Re number increases. Otherwise, the minimum of the correlation function is located somewhere around *Re* > 0. For a certain range of Re number, the value of the correlation function would decrease even though Re number is increasing, resulting in mistakes in predicting entrance length. 

**Figure 14:**
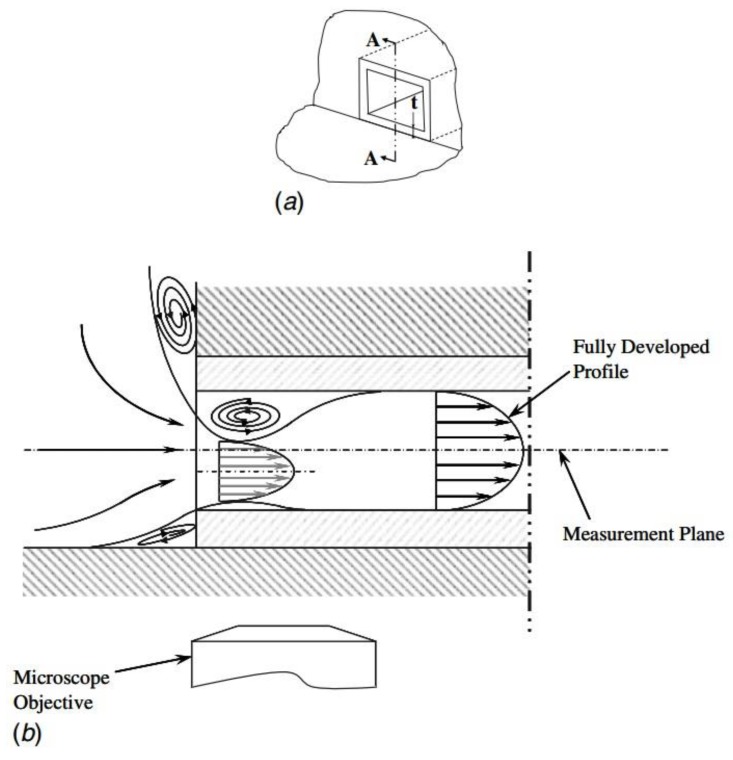
Test-section microchannel inlet configuration of Ahmad et al. [31]: (**a**) Isometric view of the microchannel entrance with the reservoir walls and (**b**) separation zone at section A-A for the microchannel entrance due to the asymmetric vena contracta effect.

It can be realized that the entrance length estimated by Equation (6) is longer than that estimated by equations proposed in this work. This disparity may due to the distinction of inlet configuration of the microchannels between Ahmad et al. [31] and this work. Figure 14 is quoted from Ahmad et al. [31]. The inlet configuration was asymmetrical in the vertical direction. The height difference between the top surfaces of the reservoir and the channel is considerable, while that between the bottom surfaces is much smaller. On account of this asymmetry, a pair of asymmetrical separation regions were generated near the inlet, shifting the velocity profile down so that the maximum velocity was not located in the measured mid-depth plane. In this work, the top and bottom surfaces of the reservoir and the channel are in the same planes, respectively. Separation in the vertical direction is avoided and the maximum velocity was located in mid-depth plane. Within the entrance region, for the same Re number and the same location away from the inlet, local velocities in the mid-depth plane measured in this work are larger than those measured by Ahmad et al. [31]. As the criterion adopted to judge if a fully developed profile is whether the local centerline velocity in mid-depth plane has attained 99% of its theoretical fully developed value, an extra distance is necessary in the experiment proceeded by Ahmad et al. [31] to shift the maximum velocity back to the mid-depth plane and continually accelerate to achieve a fully developed profile. 

## 4. Numerical Simulation

### 4.1. Investigation Method

For numerical simulation, microchannels were modeled and incompressible laminar Navier–Stokes equations were solved with ANSYS CFX 15.0. Constant fluid properties were assumed and viscous dissipation was neglected, which proved to be valid for water flow in microchannels with hydraulic diameters larger than 100 μm [34,35,36].

Three different models were constructed. Model 1 was based on the microchannel tested in former experiments of this work, which consisted of a reservoir and a channel. Model 2 was investigated by Galvis et al. [37], where only the channel was modeled and a correlation for a square cross-section channel based on numerical simulation with Model 2 was proposed by Galvis et al. [37] as

(9) $$\frac{L_{e}}{D_{h}}=\frac{0.74}{0.09Re+1}+0.0889Re.$$

**Figure 15:**
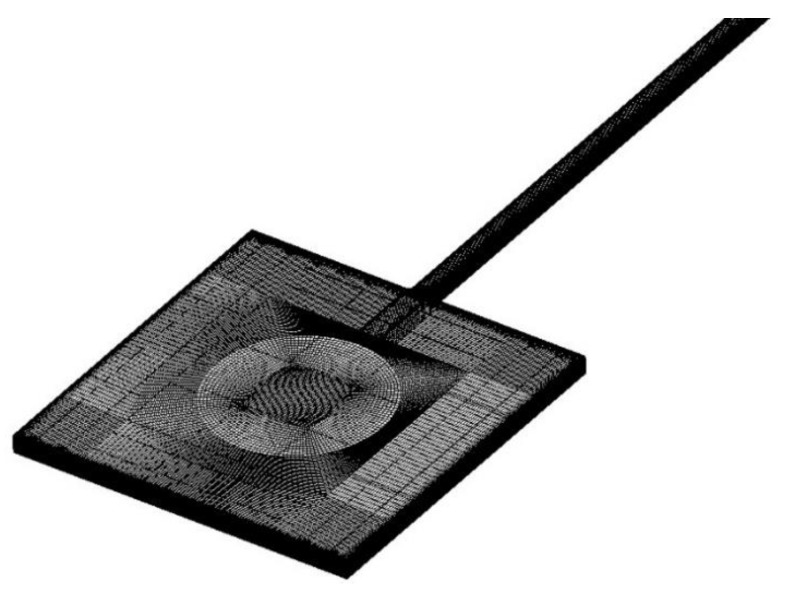
Computational domain for Model 1.

Model 3 was familiar to Model 1 and to research the difference in entrance length caused by the inlet, a right-angled inlet was adopted in Model 3, resulting in uniform velocity distribution at the inlet for microchannels of different hydraulic diameters. The computational domain for Model 1 is illustrated in Figure 15. For both models, no-slip boundary conditions were set at the inner surface of walls. At the inlet, a massflow condition was applied while a static pressure of 0 Pa was set at the outlet. Structured mesh was adopted and the mesh was refined near the walls. 

The grid convergence index (GCI) proposed by Roache [38] was employed to proceed with the mesh independence studies. The estimated error (*ε*) referred in GCI was defined as

(10) $$\varepsilon =\left[\frac{\Delta p_{f}-\Delta p_{c}}{\Delta p_{c}}\right]\frac{1}{r^{m}-1},$$

where $\Delta p_{f}$ represents the pressure drop calculated with the fine grid, while $\Delta p_{c}$ is the equivalent calculated with the coarse one, *r* means the grid expansion factor, and m refers to the order of accuracy. For a 100 μm channel when *Re* = 5, grid convergence results for model 1 are shown in Table 3. Based on this mesh independence study, mesh configuration 4 was employed and the same process was applied for all the channels and Re numbers.

### 4.2. Results and Discussion

**Figure 16:**
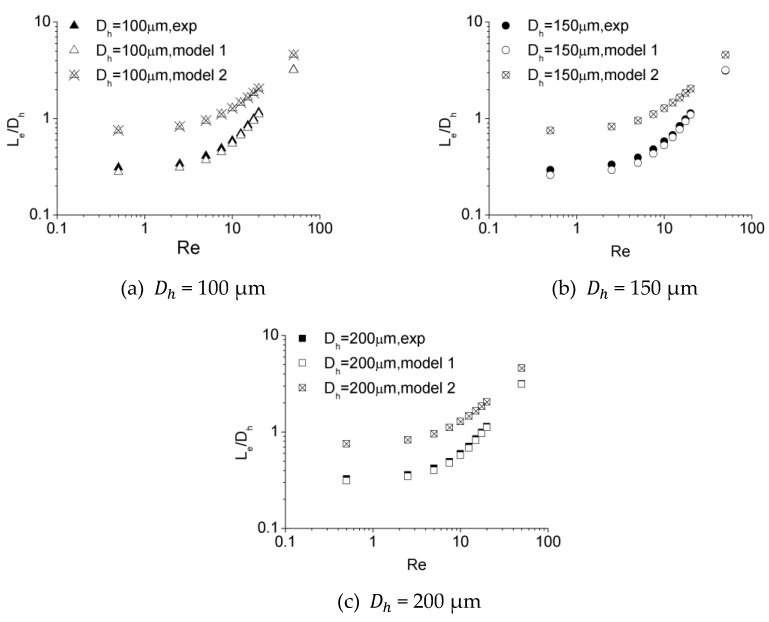
Comparison of entrance length from experiments and simulations for (**a**) 100 μm, (**b**) 150 μm, and (**c**) 200 μm.

**Figure 17:**
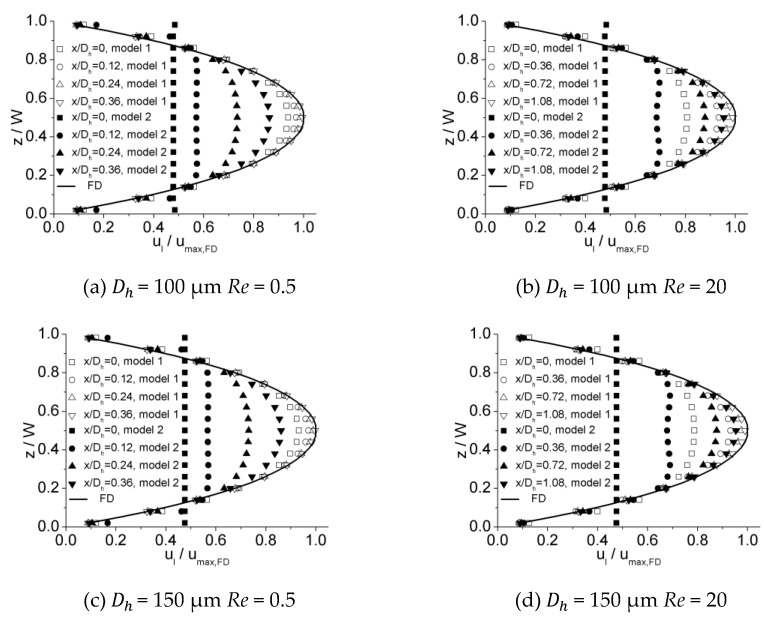

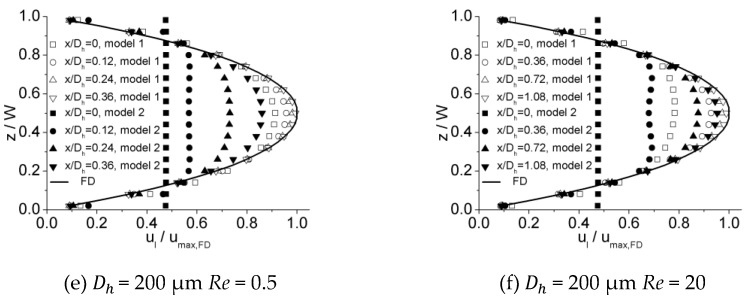
Comparison of developing velocity profiles near and downstream of the inlet for Model 1 and Model 2. (**a**) *Re* = 0.5 for 100 μm, (**b**) *Re* = 20 for 100 μm, (**c**) *Re* = 0.5 for 150 μm, (**d**) *Re* = 20 for 150 μm, (**e**) *Re* = 0.5 for 200 μm, and (**f**) *Re* = 20 for 200 μm.

Figure 16 illustrates the dimensionless hydraulic entrance length ($L_{e}/D_{h}$) versus the Re number for experimental data obtained in this work as well as numerical simulation results of Model 1 and Model 2. It can be observed that there is a strong agreement between the experimental data and the estimations of Model 1 within an error of 7.2%, while Model 2 tends to overestimate the entrance length. This disparity is caused by the different velocity distribution at the inlet due to different inlet configurations for these two models. The developing velocity profiles near and downstream of the inlet for Model 1 and Model 2 are shown in Figure 17. The plots show that the velocity distribution near the inlet for Model 1 are more approximated to experimental data, while those for Model 2 tend to be unified due to inlet configuration at the inlet. This distinction in initial velocity distribution impacts the velocity profiles downstream significantly. For different downstream locations along the length, the velocities near the center of channel for Model 1 are larger, resulting in a shorter distance to obtain a fully developed profile compared with Model 2. The agreement between the experimental and numerical data proves that scale effect is insignificant for flow in microchannels tested in this work and, with refined grid, conventional Navier–Stokes equations and numerical simulation methods can be applied in predicting laminar flow characteristics in these microchannels.

**Figure 18:**
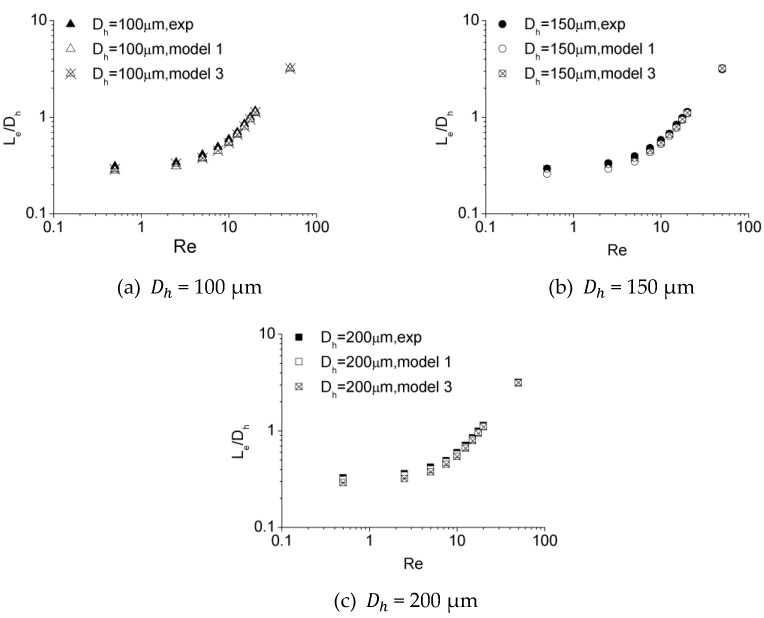
Comparison of entrance length from experiments and simulations for (**a**) 100 μm, (**b**) 150 μm, and (**c**) 200 μm.

**Figure 19:**
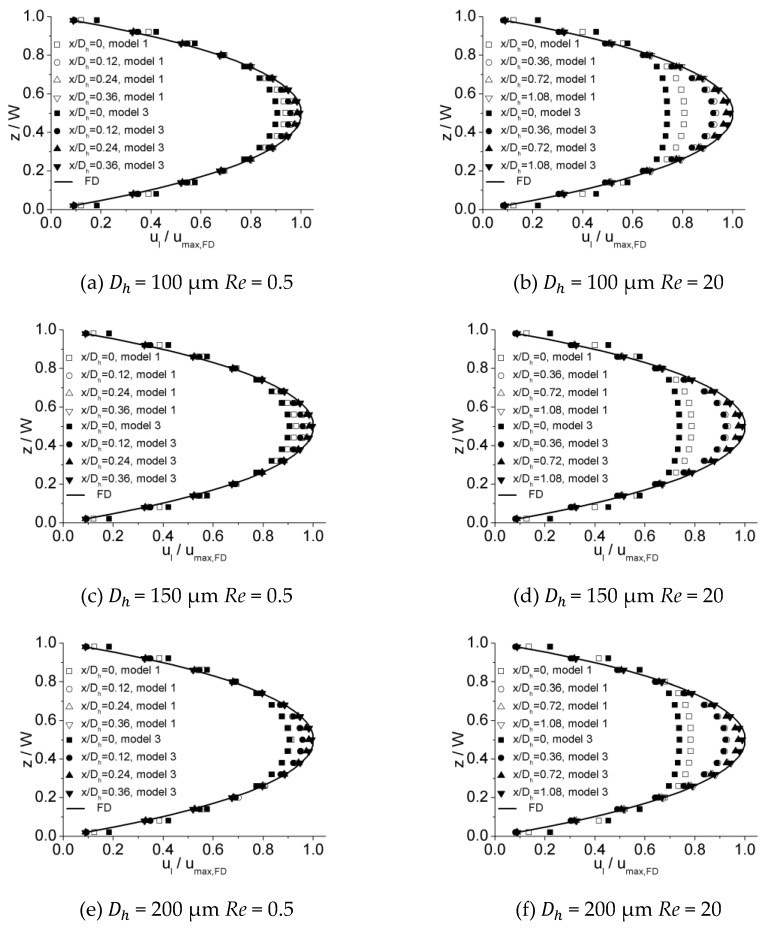
Comparison of developing velocity profiles near and downstream the inlet for Model 1 and Model 3. (**a**) *Re* = 0.5 for 100 μm, (**b**) *Re* = 20 for 100 μm, (**c**) *Re* = 0.5 for 150 μm, (**d**) *Re* = 20 for 150 μm, (**e**) *Re* = 0.5 for 200 μm, and (**f**) *Re* = 20 for 200 μm.

Figure 18 illustrates the dimensionless hydraulic entrance length ($L_{e}/D_{h}$) versus the Re number for the numerical simulation results of Model 1 and Model 3. It can be noted that the results of these two models are in agreement, indicating that the impact of inlet configuration is insignificant for Model 3 and Model 1. The developing velocity profiles near and downstream of the inlet for Model 1 and Model 3 are shown in Figure 19. For lower Re numbers the developing velocity profiles are approximated and for higher Re numbers differences can be noted near the inlet. These diminish in the region downstream of the inlet and the impact of this difference on hydraulic entrance length is insignificant. The dimensionless hydraulic entrance length of Model 3 for all these three microchannels tends to be uniform. A correlation for the results of Model 3 can be given as

(11) $$\frac{L_{e}}{D_{h}}=\{\begin{array}{c} \frac{0.28}{0.1Re+1}+0.04Re\;(Re<12.5)\\ \;0.0578Re\;(15<Re<50) \end{array},$$

which is similar to the correlation drawn from the results of experiment (Equation (8)), implying that the deviation caused by the distinction in the inlet configuration for Model 3 and Model 1 is acceptable.

## 5. Conclusions

Both experimental and numerical investigations were conducted to study flow characteristics within a hydrodynamic entrance region in microchannels with a square cross-section for laminar flow, over a Reynolds number range of 0.5 to 50. Three channels with hydraulic diameters of 100 μm, 150 μm, and 200 μm were investigated and no scale effect was observed. The influence of inlet configuration on hydrodynamic entrance length was discussed.

New correlations which are more applicable in microdevices have been proposed to estimate hydrodynamic entrance length based on Reynolds number for tested channels and strong agreement between experimental data and correlations is achieved. There is a nonlinear relationship between hydrodynamic entrance length and the Reynolds number for *Re* < 12.5, which then converts into a linear relationship as the Reynolds number increases (*Re* > 15). The Re number ranging from 12.5 to 15 is considered as a transition region, where both nonlinear and linear correlations could be applied. For investigated microchannels and the Reynolds number range compared with conventional correlations, a noticeable difference is observed for lower Re numbers (*Re* < 12.5) and good agreement is found for higher Re numbers (*Re* > 15). The results of the experiments show that, as Re number increases from 0.5 to 5 by 10 times, the dimensionless hydrodynamic entrance length increases from approximately 0.32 to 0.42 by only 30%, while for *Re*→0, dimensionless hydrodynamic entrance length is still a finite value. This may be ascribed to the lower amount of sheer stress resulting from the gentler velocity gradient under these conditions. Comparison between the results of this work and previous research shows that deviation from symmetrical velocity distribution due to inlet configuration can significantly affect hydrodynamic entrance length. This impact should be taken into consideration in the design and fabrication of microdevices.

## Figures and Tables

**Table 1:** Coefficients in Equation (1) and Equation (2).

Correlations	*A*	*B*	*C*
Atkinson et al. [19]			
Tube	0.590	0.056	–
Parallel plates	0.625	0.044	–
Chen [20]			
Tube	0.600	0.035	0.056
Parallel plates	0.630	0.035	0.044

**Table 2:** Conclusions of some previous experimental research on entrance region in microchannels with microscopic particle image velocimetry (micro-PIV).

Reference	Size of Microchannel	Range of Re Number	Conclusions
Zhang et al. [27]	50–254 μm in diameter	$\sim 10^{-5}–10^{-2}$	Velocity distribution measured by micro-PIV was in strong agreement with the value calculated by the Navier–Stokes equation.
Lee et al. [28]	690 μm in height 260 μm in width	~250–2100	Fluid had been pre-developed in pipe before entering the microchannel, causing a shorter entrance length.
Lee and Kim [29]	58 μm in depth 100 μm in width	1	Entrance length for microchannels is much smaller compared with conventional channels.
Hao et al. [30]	237 μm in hydraulic diameter	~50–1200	$\frac{L_{e}}{D_{h}}=0.08–0.09Re$
Ahmad and Hassan [31]	100, 200, and 500 μm in hydraulic diameter	~0.5–200	$\frac{L_{e}}{D_{h}}=\frac{0.6}{0.14Re+1}+0.0752Re$

**Table 3:** Grid convergence index (channel 100 × 100 μm and *Re* = 5).

Mesh No.	Nodes	Elements	Δp, Pa	*r*	*ε*
1	1147384	1066770	2270.29	-	-
2	1582848	1485551	2269.04	1.39	−0.059%
3	2025528	1918673	2267.84	1.29	−0.079%
4	2518888	2401230	2266.97	1.25	−0.068%
5	3242004	3126872	2266.28	1.30	−0.043%
